# Relating mathematical abilities to numerical skills and executive functions in informal and formal schooling

**DOI:** 10.1186/s40359-022-00740-9

**Published:** 2022-02-11

**Authors:** Peera Wongupparaj, Roi Cohen Kadosh

**Affiliations:** 1 Cognitive Science and Innovation Research Unit, College of Research Methodology and Cognitive Science, Burapha University, Saen Suk, Thailand; 2 Department of Experimental Psychology, University of Oxford, Oxford, UK

**Keywords:** Domain-specific early mathematics, Number-specific executive functions, Mathematical abilities, Preschool and primary school children

## Abstract

**Background:**

The current evidence on an integrative role of the domain-specific early mathematical skills and number-specific executive functions (EFs) from informal to formal schooling and their effect on mathematical abilities is so far unclear. The main objectives of this study were to (i) compare the domain-specific early mathematics, the number-specific EFs, and the mathematical abilities between preschool and primary school children, and (ii) examine the relationship among the domain-specific early mathematics, the number-specific EFs, and the mathematical abilities among preschool and primary school children.

**Methods:**

The current study recruited 6- and 7-year-old children (*N*_total_ = 505, *n*_6yrs_ = 238, and *n*_7yrs_ = 267). The domain-specific early mathematics as measured by symbolic and nonsymbolic tasks, number-specific EFs tasks, and mathematics tasks between these preschool and primary school children were compared. The relationship among domain-specific early mathematics, number-specific EFs, and mathematical abilities among preschool and primary school children was examined. MANOVA and structural equation modeling (SEM) were used to test research hypotheses.

**Results:**

The current results showed using MANOVA that primary school children were superior to preschool children over more complex tests of the domain-specific early mathematics; number-specific EFs; mathematical abilities, particularly for more sophisticated numerical knowledge; and number-specific EF components. The SEM revealed that both the domain-specific early numerical and the number-specific EFs significantly related to the mathematical abilities across age groups. Nevertheless, the number comparison test and mental number line of the domain-specific early mathematics significantly correlated with the mathematical abilities of formal school children. These results show the benefits of both the domain-specific early mathematics and the number-specific EFs in mathematical development, especially at the key stages of formal schooling. Understanding the relationship between EFs and early mathematics in improving mathematical achievements could allow a more powerful approach in improving mathematical education at this developmental stage.

## Introduction

Mathematical skills are regarded as an important tool and an integral part of effective functioning in everyday life [1, 2]. These skills are the keys to analyzing and interpreting information and also making basic or complex decisions [3]. Meanwhile, several lines of evidence show that early mathematics achievement might predict a person’s professional success and economic growth [4, 5]. Understanding these developmental trajectories and cognitive underpinnings is essential because of the promising predictability of later positive outcomes as a result of early numerical abilities.

Researchers from several disciplines have currently begun to reveal underlying cognitive and brain architectures of our numerical processing abilities (e.g., [6–11]). One theoretical perspective explains the diversity in early numerical ability by referring to the development of the domain-specific approach. The theory states that early numerical abilities and difficulties are closely related to numerical core systems (i.e., the approximate number system [ANS]; [12, 13]). Typically, several studies on infants and young children suggested progressive acquisitions of numerical development [14–16], where the ANS nonsymbolic and symbolic numerical magnitude processing abilities (as indexed by dot–dot and dot–number comparison and a mental number line tests) were assumed to form the basis of numerical skills among preschoolers who had not yet been taught a formal mathematics lesson [12]. A more accurate ANS or symbolic magnitude comparison ability (e.g., a number comparison test) and symbolic magnitude estimation ability (e.g., the mental number line test) were later developed [17, 18]. This continuing numerical ability uses the prenumerical ANS and is also being thought of as the numerical development from subitizing, counting, and estimating to arithmetic [16]. These early numerical systems have also been called the “core-systems of number” [12].

Other studies have also alternatively revealed the main roles of executive functions (EFs) [19] as a crucial predictor of early numerical abilities. This domain-general theoretical framework proposes that symbolic numerical magnitude estimation ability, as measured by the mental number line test, may need more than complete core systems of number [20, 21]. It has been proposed that this process includes broad cognitive processes, such as EFs, that work together with numerical processing to influence numerical development throughout childhood [22]. EFs in early childhood show significant improvements after age 5, demonstrated in abilities such as shifting (cognitive flexibility), inhibition of dominant or prepotent responses, and updating of working memory [23–26]. The majority of the research suggests that domain-general skills contribute to early numerical development, especially during the transition from preschool to kindergarten [27, 28]. Nonetheless, several contradictory results on the contribution of domain-general abilities to numerical development and the processes driving their integration remain uncertain. [29]. Specifically, the how, why, and what components of EFs in numerical context are essential and whether or not EFs are genuinely malleable to leverage early mathematical development from informal to formal schooling [30, 31].

A multicomponent framework of mathematics highlighting the main role of EFs on domain-specific numerical skills and early numerical abilities as being an indirect and stable relationship from age 8 years through to young adults has been documented [31]. This study did not capture the early stage of informal mathematical growth. The unique contributions of either the general EFs or EFs in numerical contexts on domain-specific numerical skills and early numerical abilities across age groups are also unclear [32]. A recent study found that general EFs skills did not affect mathematics achievement across age and grade (preschool–fourth grade) [33], but a recent longitudinal finding suggested that only EFs in a numerical context were far more important than ANS or general EFs to predict developmental dyscalculia and numerical accomplishment [34]. This finding is consistent with prior research that discovered a significant contribution of EFs-related numerical, but not non-numerical, content to mathematical abilities in 93 children [35]. Further, only EFs in numerical context, beyond general EFs, could predict developmental dyscalculia and mathematics achievement from ages 4 to 13 [34].

More specifically, the general EFs or EFs in numerical contexts may consist of partially dissociable components in early childhood [36–40]. The numerical specific EFs or EFs in numerical contexts have a stronger link to children’s math growth over and above the general EFs [40] because children’s ability to attend to numerical and spatial magnitudes involving in mathematics achievement may differ from those of music activities or reading counting books [41].

Several studies demonstrated the significant connection among specific executive functioning, that is, working memory, inhibition and shifting abilities, and mathematical ability in children [35, 42]. Inhibitory control is required to inhibit a dominant or irrelevant response [43]. Working memory refers to the ability to hold, update, and manipulate information within memory storage [25, 43]. Shifting ability is the ability to switch attention between tasks, mental sets, and strategies or the ability to flexibly disengage or engage with specific parts within tasks [25, 39]. Nonetheless, the relationship between EF components and mathematical competencies in early childhood may depend on some aspects of EF and specific mathematical concepts (i.e., early numeracy, counting, conceptual, and procedural skills) [44, 45].

The developmental patterns for the relationship between inhibitory control and shifting/switching abilities with mathematics achievement differed depending on the academic outcomes examined [46]. That is, few developmental changes were shown in the connection between EF components and mathematical abilities across elementary schools [42]. The unique contribution of EF components on mathematics outcome has also not been fully understood because the key roles of EF components on mathematics achievement were discovered, particularly for older students [47]. There is little research that highlights the interface of emerging and specific EF components and early mathematical learning in preschool children beyond numeracy and counting to emergent and critical mathematical proficiency in primary school children [45, 48]. These works have mainly relied on general EF assessment even though extensive research has been carried out in young children on the EF components and mathematics achievement in several contexts [42, 49, 50].

Although recent literature support transfer effects from EF interventions to mathematical abilities, the EF trainings have a larger effect in preschool than in school ages [51–53]. Further, some studies suggested ineffective transfers from EF trainings to mathematics outcomes [50, 54]. It is possibly suggested that previous approaches might fail to consider the specific ways in which EF is related to mathematics [55]. The lack of training transfer could stem from the fact that number-specific EFs are more significantly correlated with mathematical abilities than EFs measured by tasks that do not involve numerically relevant stimuli [34, 56–58]. Taken together, these pieces of information appear to imply that the heterogeneity of general EF measures may mask the relationship between EF components and mathematical achievement [46, 56]. Therefore, the EFs-numerical contexts tasks should be emphasized and used to investigate the complex relationship between specific components of the EFs and mathematics achievement.

**Fig. 1:**
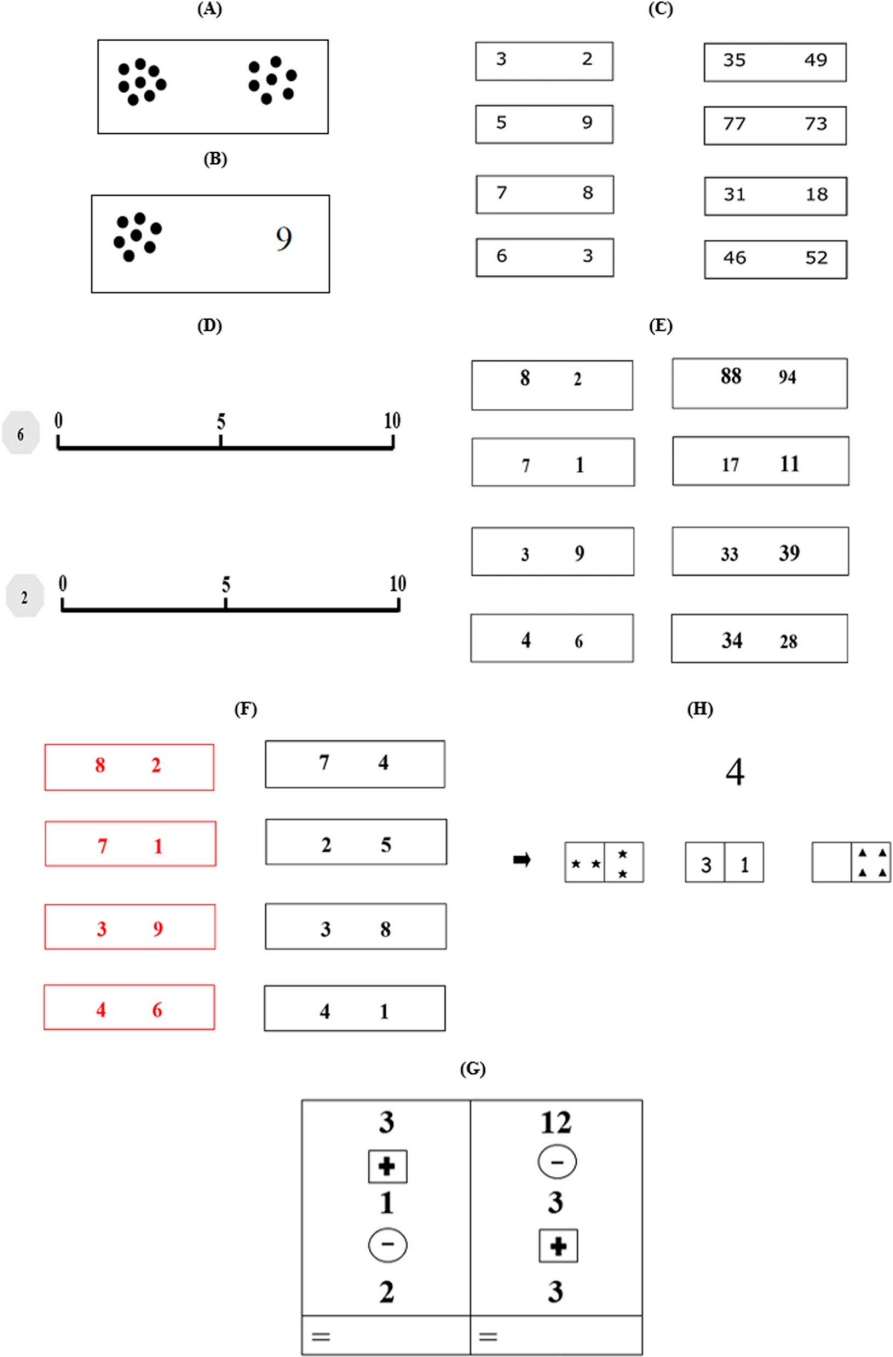
Screenshots of the tests in the current study: **A** The dot-dot comparison test; **B** The dot-number comparison test; **C** The number comparison test; **D** The mental number line; **E** The numerical Stroop test; **F** The numerical shifting test; **H** The number sets test; **G** The numerical operation test

It can be concluded that several studies have focused on numerical and domain-general executive functioning skills [59, 60]. The primary functions of the domain-specific early mathematical skills (i.e., ANS) and number-specific EFs (i.e., EFs in numerical contexts) from informal to formal schooling are relatively scarce. Such knowledge could shed light on the development of mathematical achievement in that the number-specific EF was conceptualized as the main underlying processes or mechanisms for driving the domain-specific early mathematics across the development of numerical cognition in children. The current investigation aimed to (a) compare the domain-specific early mathematics, the number-specific EFs, and the mathematical abilities between preschool and primary school children and (b) examine the relationship among the domain-specific early mathematics, the number-specific EFs, and the mathematical abilities among preschool and primary school children. Structural equation modeling (SEM) was employed to test the direct and indirect effects of the domain-specific early mathematics and number-specific EFs on mathematical abilities among preschool [6 years old] and primary school (7 years old) children. Domain-specific early mathematics were categorized by the dot-dot comparison, the dot-number comparison, the number comparison, and the mental number line tests. Number-specific EFs were represented by the numerical inhibitory and the numerical shifting tests. Formal and informal mathematical abilities were measured by the number sets [61] and the numerical operation tests (Fig. 1).

## Methods

### Participants

The current study included 511 6- to 7-year-old children (238 or 47.1% for 6-year-old preschoolers and 267 or 52.9% for 7-year-old first graders); six children were excluded because of missing values, thus leaving 505 children (50.2% female participants) for final analysis. All participants were native Thai and attended 12 public schools in Chonburi province, Thailand and a sample of 12 public schools was drawn using a stratified sampling technique. All public schools used the same set of subjects and standards under the national curriculum for Thailand. The preschoolers and first graders were studying at the same public schools with equal proportions. No participant was clinically referred for learning difficulties (LD) or attention-deficit/hyperactivity disorder (ADHD). The experimental protocols were approved by the Burapha University Research Ethics Committee (BUU 6200/01533). All methods were carried out in accordance with the Good Clinical Practice (GCP) guidelines and the Declaration of Helsinki. Written informed consent was obtained from parents of all participants prior to inclusion.

### Measures

The domain-specific early mathematics is composed of eight paper-and-pencil tests (the dot-dot comparison test, the dot-number comparison test, the number comparison test [also termed symbolic magnitude processing test [1], and the mental number line], the number-specific EFs (numerical inhibitory and shifting tests), and the mathematical abilities (number sets and numerical operation tests). These domain-specific early mathematics assessments were developed to tap into distinct aspects of young children’s mathematics development that are considered to be essential in preschool and primary school mathematics [18, 20]. The number-specific EFs measures were used to reflect common and specific aspects of EFs in numerical contexts [62]. These tests were chosen to fit best with the age range tested in the current study and fit the emerging literature on the structure of EFs in the preschool and primary school periods [56, 58, 63].

The number sets and numerical operation tests were selected to index the mathematical abilities because these measures were used to reflect different mathematical skills rather than mathematics achievement in a single multicomponent standardized test [61, 64]. The number sets test was designed to assess a small area of early numeracy or “number sense” and fluency in identifying and processing quantities indexed by object sets and numerals [61, 65], whereas the numerical operation test was used to measure basic arithmetic skills or “arithmetic fact” in line with the national curriculum for preschool and primary school levels. These tests were administered in quiet rooms that were provided by the schools, and a group-administered test was used for all children at their schools. All children were not allowed to count and/or take notes, and these behaviors were monitored by researchers. The constructs, tests, test lengths, and time spent are shown in Table 1. All children were assessed across eight tests, and the test administration took approximately 33 min for each child.

**Table 1:** The constructs, tests, test length, and time spent

Constructs		Tests	Number of items	Time spent (min)
The domain-specific early mathematics	Dot-Dot comparison	Dot-Dot comparison test	30	2.5
	Dot-Number comparison	Dot-Number comparison test	30	2.5
	Number comparison	Number comparison test (Single & Double digits)	60 (Single) 60 (Double)	2 3
	Analogous Magnitude representation	Mental number line: Percent Absolute Error	10	5
The number-specific EFs	Inhibition	Numerical Stroop test (Single & Double digits)	30 (Single) 30 (Double)	2 3
	Shifting	Numerical shifting test	35	3
Mathematical abilities		Number sets test	16 (5 set) 16 (9 set)	2 3
		Numerical operation test	20	5

#### The dot-dot comparison test

The dot-dot comparison test was used to assess the enumerating ability by comparing two sets of dots that reflect subitizing and counting systems of children’s early numerical abilities [66]. The dot-dot comparison test is composed of 30 items, and each item contains two sets of black dots with a pseudorandom arrangement on a white background (see Fig. 1A). The average distance between the centers of the two black-dot sets was 2.93 cm (minimum = 2.80 cm and maximum = 3.0 cm). Each dot was equated in size (0.30 cm in diameter), each group of dots was also comparable in size (1.00 cm in diameter), and numerosity (several dots from 1 to 9) differed across items. All children were instructed to circle which set of dots contained more dots without counting as accurately and quickly as possible within 2.5 min. A response was scored as correct (1 point) and incorrect (0 point) with a range of scores between 0 and 30. The correct answer for each item was counterbalanced, and no more than three consecutive right answers on the same side were shown [1]. The Kuder–Richardson (KR)-20 reliability coefficient of this test was 0.97.

#### The dot-number comparison test

The dot-number comparison test was used to assess the numerical ability in associating and comparing a perceived number of objects (dots) with Arabic numerals (nonsymbolic vs. symbolic numbers). An Arabic symbolization is required for the development of the mental number line as a representation of magnitude and ordinality to visual space [67]. The dot-number comparison test contained 30 items, and each item contains two different sets of black dots and a single digit presenting on a white background with a pseudorandom arrangement on the left side and the single digit on the right side (see Fig. 1B). The mean distance between the centers of the dot-number pairs was 2.99 cm (minimum = 2.9 cm and maximum = 3.2 cm). All dots were equated in size (0.3 cm in diameter), each group of dots was also equal in size (1 cm), and several dots ranged from 1 to 9. All the single digits were displayed in 20-point Times New Roman font. All children were instructed to circle which of the two sets between the dot–number pair is larger without counting as accurately and quickly as possible within 2.5 min. A response was scored as correct (1 point) and incorrect (0 point) with a range of scores between 0 and 30. The correct answer for each item was also counterbalanced, and no more than three successive correct answers on the same side were shown [1]. The KR-20 reliability coefficient of this test was 0.97.

#### The number comparison test

The number comparison test was used to examine symbolic numerical magnitudes [1]. The number comparison test is composed of two numerical magnitude comparison subtests: a single-digit subtest with digits ranging from 1 to 9 and a two-digit subtest with digits between 11 and 99. The 120-digit pairs (60 pairs for single and 60 pairs for two-digit subtests) were displayed in four columns of 15 pairs in a 12-point Verdana font for each subtest (see Fig. 1C). The number pairs were randomly presented, and four factors were taken into account: (1) a counterbalance of the correct answer on the side in each column, (2) different numbers in subsequent or neighboring number pairs, (3) no more than three consecutive correct answers presenting on the same side, and (4) no similar or inverse number pairs (e.g., 6–2 vs. 2–6) presenting in the same row or column. All children were instructed to circle the larger of the single or two-number pairs as accurately and quickly as possible within 2 and 3 min for single- and two-digit subtests. A response was also scored as correct (1 point) and incorrect (0 point) with a range of scores between 0 and 60 for both single- and two-digit subtests. The KR-20 reliability coefficient of this test was 0.99 for the single-digit subtest, 0.98 for the two-digit subtest, and 0.99 for the overall numerical comparison test.

#### The mental number line test

The mental number line test was used to assess proficiency in numerical magnitude processes and representations [68]. The mental number line test contained 10 items, and all children were instructed to estimate by crossing out a location of 10 target numbers on 13-cm number lines. Each horizontal number line started with a target number and a 0 at the left endpoint and numbers (i.e., 10, 20, 50, and 100) at the right endpoint (see Fig. 1D). All digits were displayed in a 12-point and 16-point Times New Roman font for target numbers and anchored numbers at the left and right endpoints of the mental number line test, respectively. They were instructed to complete the test as accurately and quickly as possible within 5 min. A response was scored in line with the percent absolute error (PAE) formula [21] and was defined as the absolute difference between target number and children’s estimate divided by the scale of each item and expressed as a percentage (i.e., |target number − participant’s estimated number|]/numerical range) × 100. The PAE scores ranged from 0 to 100%, and a higher PAE score indicated a less accurate series of estimates. The internal consistency with Cronbach’s *α* was 0.77.

#### The numerical inhibition test

The numerical inhibition test was used to assess a cognitive inhibition or the ability to automatically inhibit irrelevant responses and adjust control [69–71] on physical and numerical pairs. The numerical inhibition test contained two subscales, that is, a one-digit subtest with digits ranging from 1 to 9 and a two-digit subtest with digits ranging from 11 to 99. The 60-digit pairs (30 pairs for single and 30 pairs for two-digit subtests) were displayed in three columns of 10 pairs in 22-point and 26-point Times New Roman font for smaller and larger physical sizes. The distances between two digits of each number pair were six, four, and two for the first, second, and third columns, respectively (e.g., 1 7, 2 6, and 3 5; see Fig. 1E). The number pairs were randomly shown, and four factors were also taken into consideration: (1) a counterbalance of the right answer on the side in each column, (2) different numbers in subsequent or neighboring number pairs, (3) no more than three consecutive correct answers showing on the same side, and (4) no similar or inverse number pairs (e.g., 1 5 vs. 5 1) presenting in the same row or column. All children were instructed to compare the physical sizes of two numbers and circle the larger of the single or two-number pairs as accurately and quickly as possible within 2 and 3 min for single- and two-digit subtests. A response was scored as correct (1 point) and incorrect (0 point) with a range of scores between 0 and 30 for both single- and two-digit subtests. The KR-20 reliability coefficient of this test was 0.98 for the single-digit subtest, 0.95 for the two-digit subtest, and 0.98 for the overall test.

#### The numerical shifting test

The numerical shifting test was used to assess children’s cognitive flexibility performance or the ability to shift attention on the basis of changing (numerical) condition demands [72]. The paper-and-pencil version for the children was adapted from the computerized switching task by modifying the procedures and stimuli [73, 74]. The numerical shifting test contained 36 items with digit pairs ranging from 1 to 9. The 36-digit pairs were showed in three columns of 12 pairs in 26-point Times New Roman font for each column. The digit pairs were displayed in red or black: the red digit pairs signaled to the children that it was a greater-than-five condition and the black digit pairs indicated that it was an odd–even condition. Each column is composed of three-set shifts between greater-than-five and odd–even conditions (see Fig. 1F). The number pairs were randomly displayed, and four factors were also taken into consideration: (1) a counterbalance of the correct answer on the side in each column, (2) different numbers in subsequent or neighboring number pairs, (3) no more than three consecutive correct answers showing on the same side, and (4) no similar or inverse number pairs (e.g., 5 2 vs. 2 5) presenting in the same row or column. All children were instructed to decide which red digit is greater than five and which black digit is odd or even as accurately and quickly as possible within 3 min. A response was scored as correct (1 point) and incorrect (0 point) with a range of scores between 0 and 36. The KR-20 reliability coefficient of this test was 0.95.

#### The number sets test

The number sets test was used to assess mathematical abilities in young children [64]. The number sets test is composed of 32 items with 16 items for each target number: “five” and “nine.” Each item contained a pair or trio of Arabic numbers with an 18-point font in a half-inch square, object sets (stars, circles, diamonds, and triangles) in a half-inch square, or both of them, and the Arabic numbers and object sets were combined to create domino-like rectangles (see Fig. 1G and further details in a previous study [61]). All children were instructed to circle any groups that can be put together to make the number at the top of the page, which could be 5 or 9, and to complete as quickly as possible within 2 and 3 min for the targets “5” and “9”, respectively. A response was scored as correct (1 point) and incorrect (0 point) with a range of scores between 0 and 16 for the targets “5” and “9” and between 0 and 32 for both targets. The KR-20 reliability coefficient for the targets “5” and “9” were 0.94 and 0.95 and 0.96 for both targets.

#### The numerical operation test

The numerical operation test was adapted and used to assess children’s storage and manipulation of numerical operations [75, 76]. This test was also called “arithmetic facts” in the literature, but it included only addition and subtraction in basic forms. The test items were reviewed, and all items were consistent with education and curriculum in preschool and primary school levels. The numerical operation test is composed of 20 items: 8 items for single-digit numerical operations and 12 items for double-digit numerical operations. The 20 items of numerical operations were shown in four columns in 22-point Times New Roman font for each column (see Fig. 1H). All children were only asked to write down the answer as the outcome of numerical operations such as adding and carrying. A response was scored as correct (1 point) and incorrect (0 point) with a range of scores between 0 and 20. The KR-20 reliability coefficient of this test was 0.95.

### Statistical analysis

MANOVA was used to evaluate the age group differences between preschool (6 years old) and primary school (7 years old) children across eight dependent variables to answer the research questions and test the research hypotheses. The partial *η*^2^ was also calculated to represent the magnitude of difference between groups [77, 78]. The first latent variable for domain-specific early mathematics was obtained from four observed variables, that is, dot-dot, dot-number, number comparison, and mental number line, and the second latent variable for number-specific EFs was generated from two observed variables, namely numerical inhibition and shifting, in measurement and structural models. The third variable for the mathematical abilities was also derived from two observed variables, that is, the number sets and the numerical operation. Finally, the direct paths among the first latent variable, the second latent variable, and the third latent variable were estimated.

No missing value was found for the current study. Data analyses were carried out using IBM SPSS statistics for Window, version 26 (IBM Corp., Armonk, NY, USA) and SPSS Amos version 26.0 [79–81]. The structural equation model (SEM) parameters were estimated by using the maximum likelihood procedure. The goodness-of-fit indices of the estimated models were evaluated using five indicators, that is, the *p* value of chi-square (*χ*^2^) above 0.05 and *χ*^2^/*df* smaller than 3 are preferred, the *p* value of root mean square error of approximation (RMSEA) lower than 0.07 indicates a well-fitting model, the comparative fit index (CFI), the goodness of fit index (GFI), and the adjusted GFI; the values over 0.90 suggest a good fit [82, 83]. The models for the overall pooled, 6-year-old, and 7-year-old children supported the empirical data and provided good model fits, the p values of *χ*^2^ = 0.93, 0.05, and 0.57; *χ*^2^/*df* = 0.93, 1.97, and 0.83; RMSEA =  < 0.05, 0.06, and < 0.01; CFI = 1.00, 0.99, and 1.00; GFI = 1.00, 0.98, and 0.99; Adjusted GFI = 0.98, 0.93, and 0.97, respectively.

## Results

### Descriptive statistics, group difference, and correlation coefficients among variables

Table 2 shows the domain-specific early mathematics represented by four variables, that is, dot-dot, dot-number, number comparison, and mental number line. The number-specific EFs were indexed by two variables, namely, numerical inhibition and shifting. The mathematical abilities were also represented by two variables, that is, the number sets and the numerical operation. In general, the domain-specific early mathematics of 6-year-old children was significantly lower than that of 7-year-old children, but it was clearly shown for the number comparison test that the 6-year-old children had a lower score on the number comparison test with a large effect size than that of the 7-year-old children. Similarly, the number-specific EFs for 7-year-old children were higher; however, the effect sizes for all variables in the number-specific EFs between two age groups were moderate. The mathematical abilities were better for 7-year-old children, and a strong effect size was observed.

**Table 2:** Descriptive statistics for variables indicating the domain-specific early mathematics, the number-specific EFs and the mathematical abilities among 6-and 7-year-old children (n_6yrs_ = 238/n_7yrs_ = 267/N_overall_ = 505) and age group differences

Variable	Min–Max		Mean (SD)			*α*		
	6 years	7 years	6 years	7 years	All	6 years	7 years	All
**The domain-specific early mathematics**								
Dot-Dot comparison test	0–30	0–30	18.84 (10.12)	25.24 (6.87)	22.23 (9.13)	.97	.95	.97
Dot-Number comparison test	0–30	0–30	17.36 (9.68)	23.23 (9.13)	20.46 (9.83)	.96	.97	.97
Number comparison test	0–120	0–120	49.61 (34.60)	83.44 (30.93)	67.50 (36.79)	.99	.99	.99
Mental number line	1.5–55	0–44.6	19.37 (8.21)	15.96 (8.38)	17.57 (8.46)	.76	.67	.72
**The number-specific EFs**								
Numerical Stroop test	0–60	0–60	39.34 (17.21)	51.30 (12.94)	45.66 (16.23)	.97	.97	.97
Numerical shifting test	0–35	0–35	17.52 (9.82)	23.92 (8.85)	20.90 (9.84)	.95	.94	.95
**Mathematics abilities**								
Number sets test	0–31	0–32	4.42 (6.27)	13.98 (9.39)	9.48 (9.37)	.94	.94	.94
Numerical operation test	0–20	0–20	2.46 (4.02)	7.61 (6.23)	5.18 (5.89)	.93	.94	.95

Variable	Skewness			Kurtosis			Group differences (6 vs. 7 years old)		
	6 years	7 years	All	6 years	7 years	All	*F*(*df*)	*p*	Partial η^2^
**The domain-specific early mathematics**									
Dot-Dot comparison test	− 0.49	− 1.71	− 1.05	− 1.15	2.52	− 0.11	70.49 (1)	< .01	0.12
Dot-Number comparison test	− 0.28	− 1.26	− 0.71	− 1.13	0.39	− 0.80	49.16 (1)	< .01	0.09
Number comparison test	0.49	− 0.71	− 0.16	− 0.65	− 0.18	− 1.10	134.62 (1)	< .01	0.22
Mental number line	1.03	0.66	0.76	2.59	0.67	1.51	21.25 (1)	< .01	0.04
**The number-specific EFs**									
Numerical Stroop test	− 0.79	− 2.16	− 1.29	− 0.38	4.55	0.78	78.91 (1)	< .01	0.14
Numerical shifting test	− 0.17	− 1.12	− 0.60	− 0.95	0.86	− 0.56	59.38 (1)	< .01	0.11
**Mathematics abilities**									
Number sets test	− 0.11	0.08	0.74	− 0.96	− 1.18	− 0.72	176.49 (1)	< .01	0.26
Numerical operation test	1.44	0.42	1.07	1.09	− 1.10	− 0.14	118.35 (1)	< .01	0.19

The coefficient alpha (*α*) for all measures was generally excellent (*α* ≥ 0.90), but it was only acceptable for the mental numbe line test (*α* = 0.76). All variables were normally distributed, as measured by skewness and kurtosis (see Table 2). Table 3 shows the correlation coefficients among variables representing the domain-specific early mathematics, the number-specific EFs, and the mathematical abilities for the overall pooled children (*N* = 505) broadly demonstrating a moderate relationship, that is, the correlations between variables in the domain-specific early mathematics, the number-specific EFs, and the mathematical abilities ranged from − 0.43 to 0.64 for the number sets. Table 4 shows a moderate correlation between variables reflecting the domain-specific early mathematics, and the number-specific EFs and the number sets (− 0.30 to 0.51) and the numerical operation (− 0.31 to 0.51) were observed for 6-year-old children. A weak-to-moderate connection, on the other hand, was identified between variables indexing domain-specific early mathematics and number-specific EFs and the number sets (0.34 to 0.54) and the numerical operation (0.29 to 0.51) for 7-year-old children. The multicollinearity tests were performed using the value inflation factor (VIF), and all VIF scores for pooled children, for 6-year-old children, and for 7-year-old children were less than the threshold of 5, indicating that the multicollinearity is not a problem in current datasets [83].

**Table 3:** The correlation coefficients among variables representing the domain-specific early mathematics, the number-specific EFs, and the mathematics abilities for the overall pooled children (N = 505)

Variable	Dot-Dot comparison test	Dot-Number comparison test	Number comparison test	Mental number line	Numerical Stroop test	Numerical shifting test	Number sets test	Numerical operation test
**The domain-specific early mathematics**								
Dot-Dot comparison test								
Dot-Number comparison test	.61**							
Number comparison test	.66**	.70**						
Mental number line	− .26**	− .30**	− .40**					
**The number-specific EF**								
Numerical Stroop test	.64**	.58**	.70**	− .31**				
Numerical shifting test	.47**	.50**	.59**	− .24**	.58**			
**Mathematics ability**								
Number sets test	.45**	.48**	.64**	− .43**	.50**	.48**		
Numerical operation test	.42**	.40**	.60**	− .44**	.44**	.39**	.61**	

**p < .001

**Table 4:** The correlation coefficients among variables representing the domain-specific early mathematics, the number-specific EFs, and the mathematical abilities for the overall pooled children for 6- (lower diagonal) and 7- (upper diagonal) year-old children (ns = 238 and 267)

Variable	Dot-Dot comparison test	Dot-Number comparison test	Number comparison test	Mental number line	Numerical Stroop test	Numerical shifting test	Number sets test	Numerical operation test
**The domain-specific early mathematics**								
Dot-Dot comparison test		.35**	.47**	− .20**	.49**	.25**	.34**	.29**
Dot-Number comparison test	.74**		.63**	− .22**	.37**	.44**	.37**	.28**
Number comparison test	.68**	.69**		− .35**	.63**	.50**	.54**	.51**
Mental number line	− .22**	− .29**	− .36**		− .30**	− .20**	− .44**	− .44**
**The number-specific EF**								
Numerical Stroop test	.65**	.66**	.65**	− .23**		.42**	.40**	.35**
Numerical shifting test	.51**	.45**	.55**	− .18**	.61**		.36**	.29**
**Mathematics ability**								
Number sets test	.44**	.49**	.51**	− .30**	.44**	.49**		.43**
Numerical operation test	.43**	.44**	.51**	− .31**	.39**	.34**	.49**	

**p < .001

### SEM analyses to test the relationship among 6-year-old children (n = 238), 7-year-old children (n = 267), and the overall pooled children (N = 505)

For the SEM model of 6-year old children, the dot-dot, dot-number, and number comparison tests exerted comparable effects (*β* = 0.85, *p* < 0.01; *β* = 0.85, *p* < 0.01; *β* = 0.80, *p* < 0.01, respectively) on the domain-specific early mathematics, but the mental number line showed the lowest factor loading (*β* =  − 0.30, *p* < 0.01) on the given latent variable. Further, the numercal Stroop and the numerical shifting tests also showed similar effects (*β* = 0.79, *p* < 0.01; *β* = 0.75, *p* < 0.01, respectively) on the number-specific EFs latent variable. Both latent variables significantly and positively related (*β* = 0.67, *p* < 0.01; *β* = 0.69, *p* < 0.01) to the mathematical abilities factor as measured by the number sets and the numerical operation tests.

For the SEM model of 7-year old children, the number comparison test showed the strongest factor loading on the domain-specific early mathematics (*β* = 0.94, *p* < 0.01), followed by the dot-number comparison (*β* = 0.67, *p* < 0.01), and the dot-dot comparison tests (*β* = 0.51, *p* < 0.01), respectively. However, the mental number line demonstrated the lowest factor loading (*β* =  − 0.37, *p* < 0.01) on the given latent variable in comparison to other factor loadings in measurement model. Hence, the numerical inhibition and shifting showed comparable effects (*β* = 0.67, *p* < 0.01; *β* = 0.62*, p* < 0.01, respectively) on the number-specific EFs latent variable. Both latent variables also significantly and positively related (*β* = 0.76, *p* < 0.01; *β* = 0.77*, p* < 0.01) to the mathematical abilities factor as measured by the number sets and the numerical operation tests.

**Fig. 2:**
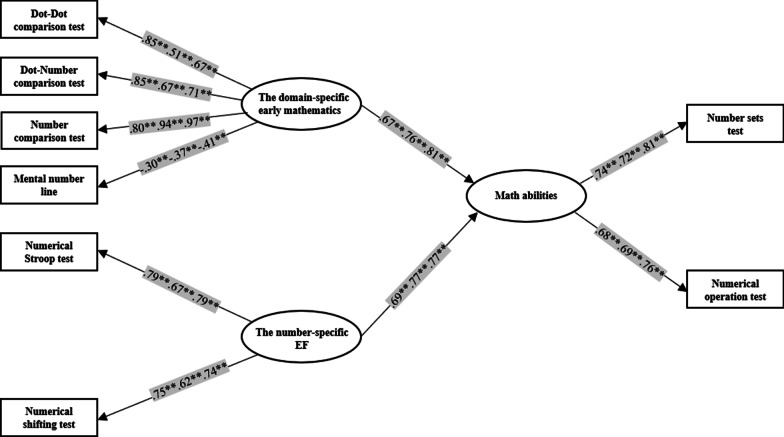
The relationships among the domain-specific early mathematics, the number-specific EF, and the mathematical abilities. Separate parameters of different age groups (6-year-old/ 7-year-old/6-and-7-year-old) and standardized coefficients (*β*s) are reported. Note **p < .01

For the SEM model of the overall pooled children, the number comparison test showed the strongest factor loading (*β* = 0.97, *p* < . 01) on the domain-specific early mathematics, followed by the dot-number comparison (*β* = 0.71, *p* < 0.01), and the dot-dot comparison tests (*β* = 0.67, *p* < 0.01), respectively. Nonetheless, the mental number line showed the weakest factor loading on the given latent variable (*β* =  − 0.41, *p* < 0.01). Hence, the numerical inhibition and shifting showed comparable effects (*β* = 0.79, *p* < 0.01; *β* = 0.74, *p* < 0.01) on the number-specific EFs latent variable. Both latent variables also significantly and positively related (*β* = 0.81, *p* < 0.01; *β* = 0.77, *p* < 0.01) to the mathematical abilities factor as measured by the number sets and the numerical operation tests (see Fig. 2).

## Discussion

The current study aims to compare and examine the effects of the domain-specific early mathematics and the number-specific EFs on the mathematical abilities in a sample of 6- and 7-year-old children. Analyses were first carried out to test the age group differences across eight dependent variables and to examine the relationships between two latent variables (i.e., the domain-specific early mathematics and the number-specific EFs) and the latent mathematical abilities in a sample of 6- and 7-year-old children.

It can be inferred from the current results that 6- and 7-year-old children (informal schooling and formal schooling) were evident on the number comparison, the number sets, and the numerical operation differences. The finding in itself shows an integrative role of numerical development among numerical comparison, storage, and manipulation abilities on mathematical achievement from preschool to primary school students [75]. The distinctive competency for the number comparison, the numerical operation, and the number sets between two age groups also suggests numerical and developmental acquisitions from understanding precise magnitudes of nonsymbolic numbers, relating nonsymbolic to a foundation of symbolic numerical representations in six-year-olds [84], to expanding understanding the small symbolic numbers to larger whole numbers (i.e., single and double digits) in 7-year-olds [16].

The dot-dot and dot-number comparison tests were used to examine the process of attributing numerical magnitude to nonsymbolic numbers in both age groups. The effect sizes of both tests were somewhat small despite the significant differences between the two age groups on both dot-dot and dot-number observed in Table 2. It is plausible that ANS acuity, nonsymbolic, and basic symbolic numerical knowledge fully reach the developmental milestone on numerical competence at younger ages [85]. This follows previous findings that demonstrated the specific effects of ANS acuity and mapping precision between numeral notations and their corresponding magnitudes that are dominant only in preschool children [86]. The performance on the mental number line test significantly differed between 6- and 7-year-old children but the extent of discrepancy was small following the literature. However, the performance in the mental number line test explained a relatively small amount of variance in the SEM model compared to the numerical comparison tasks. Although young children can count objects and understand relationships between objects and cardinal numbers, the number line further requires an understanding of lengths between the numbers written below the intervals on the number line. Thus, the number line seems to be a difficult tool to master for children who are younger than 7 and 8 years [87].

There is still a lack of agreement on the relative importance of domain-specific precursors in the development of mathematical abilities [76]. The unique contribution of the present SEM findings is the differential associations between specific indicators of the domain-specific early mathematics and the number-specific EFs and the mathematical abilities from kindergarten through primary school. The importance of subitizing, approximation, and comparison as indexed by the dot-dot and dot-number comparison tests for mathematical abilities decreased as preschool children progressed through formal schooling. Nonetheless, the symbolic and exact understanding of numerical concepts as measured by the number comparison and the mental number line tests was prioritized for the mathematical abilities with successive grades. Furthermore, the mathematical abilities were more dependent on both the domain-specific early mathematics (0.67 vs. 0.76) and the number-specific EFs (0.69 vs. 0.77) in older children. The mathematical problems will call upon a crucial process of detecting and assessing critical features of number sense [61] and involving inhibition and shifting of information [88]. A strong influence of both the domain-specific early mathematics and the number-specific EFs in older children may reflect the increasingly demanding role of shifting and inhibition capacities with age (e.g., [40, 89]).

Another main finding is that the relative importance of the domain-specific early mathematics and the number-specific EFs that are similar in size in relation to the mathematical abilities of 6-year-old children. However, the number-specific EFs showed a stronger relationship with mathematical abilities than the domain-specific early mathematics for 7-year-old children. Indeed, children are required to map and combine the different Arabic numerals and symbols onto the corresponding quantities and then compare them with the target number of each item to master mathematical competencies as measured by the number sets and the numerical operation tests. Although the present study supports the previous findings that quantity representation or ability to map quantities and magnitudes with symbols was associated with the mathematical abilities (e.g., 1, 86, 91), our results highlight the stronger association among the domain-specific early mathematics, the EFs in a numerical context, and the mathematical abilities at the beginning of formal schooling. The older children may learn school-taught mathematics, providing them with knowledge on symbol systems and procedural tools. Accordingly, to achieve mathematical calculations, the performances of EFs in a numerical context were improved in older children.

Furthermore, a more efficient supporting system or the EFs may be required to encourage the acquisition of existing early mathematical abilities and arithmetical capabilities with cumulative knowledge of symbol systems and strategy choices and discoveries in older children [90, 91]. In this view, apart from better knowledge on domain-specific early mathematics, primary school children have to rely directly on the EF subcomponents to some extent. In this case, solving mathematical problems allows the child to select relevant information or strategies, inhibiting numerical information already processed but no longer relevant. Cognitive flexibility also allows the child to switch from one strategy to another, transforming or substituting the no-longer relevant information with a new one [92–94].

Nonetheless, this study also possesses several noteworthy limitations. Given the strong link between working memory and IQ, although no children with LD and ADHD were found, the present study lacks control over children’s IQ scores. Accordingly, a cautious interpretation of the finding must be considered. In addition, working memory was found to be the second EF component to emerges, after inhibitory ability and before shifting ability, during preschool ages and it is also regarded as a tool of learning (e.g., [96, 97]). Specifically, a weakness in working memory has been documented in children with dyscalculia (e.g., [98, 99]). Thus, further study should investigate working memory as an EF component in numerical domain.

Moreover, the weak correlation among the mental number line and other variables in the same construct may additionally stem from the issues of the test format and the scoring method. A further limitation of this study is that children could have made their comparisons on the basis of continuous extent rather than the number for nonsymbolic stimuli [95, 96]. Future work needs to consider this stimulus issue for children at this young age.

For practice reasons and practices, the paper-and-pencil based tests were suitable for our settings in terms of distribution and administration of the tests. However, the response time could reflect the automatization during numerical processing of both mathematical and executive functioning skills of the young age [99]. Thus, the selection of the computerized version of these mathematical cognition tasks should also be considered for further studies because the computer-based tasks may offer several sophisticated parameters (e.g., response accuracy, response time, and difference scores), standardized test administration procedures, automatic scoring, and instant feedback for children and teachers [97–100].

The current study did not compare the relative effects of the domain-specific early mathematics, the number-specific EFs, and the general EFs on mathematical abilities. Further studies should specifically compare common and unique roles of domain-general vs. domain-specific EF on mathematical development. The present findings provide a strong motivation to delineate these factors. Finally, a longitudinal study is needed to support the current findings in regards to the differential effects of the domain-specific early mathematics and the number-specific EFs on the mathematical abilities.

## Conclusion

The present study yielded two key findings. First, 7-year-old children outperformed 6-year-old children in the overall measures of the domain-specific early mathematics and the number-specific EFs, especially for more sophisticated numerical knowledge and EF subcomponents, namely, symbolic numerical magnitude representations as indexed by the number comparison and the mental number line tests and the numerical inhibitory and shifting abilities as measured by the numerical inhibition and shifting tests. Second, both the domain-specific early mathematics and the number-specific EFs comparably and significantly related to the mathematical abilities for 6- and 7-year-old children, but the domain-specific early mathematics and the number-specific EFs were dominant concerning the mathematical abilities for 7-year-old children.

## Data Availability

The dataset for the current study is available from the corresponding author on reasonable request.

## Abbreviations

ANS: Approximate Number System
EFs: Executive Functions
LD: Learning Difficulties
ADHD: Attention Deficit Hypoeractivity Disorder
PAE: Percent Absolute Error
MANOVA: Multiple Analysis of Variance
SEM: Structural Equation Modeling
GFI: Goodness of Fit Index
VIF: Value Inflation Factor
LTM: Long-Term Memory
